# Micro-computed tomography for non-invasive evaluation of muscle atrophy in mouse models of disease

**DOI:** 10.1371/journal.pone.0198089

**Published:** 2018-05-29

**Authors:** Laura Pasetto, Davide Olivari, Giovanni Nardo, Maria Chiara Trolese, Caterina Bendotti, Rosanna Piccirillo, Valentina Bonetto

**Affiliations:** IRCCS-Istituto di Ricerche Farmacologiche "Mario Negri", Milan, Italy; University of Florida, UNITED STATES

## Abstract

Muscle wasting occurs during various chronic diseases and precedes death in humans as in mice. The evaluation of the degree of muscle atrophy in diseased mouse models is often overlooked since it requires the sacrifice of the animals for muscle examination or expensive instrumentation and highly qualified personnel, such as Magnetic Resonance Imaging (MRI). Very often behavioral tests for muscle strength evaluation are used as an outcome measurement in preclinical therapeutic trials. However, these tests are easy to perform serially, but not enough sensitive to detect early muscle changes during disease progression. Monitoring muscle loss in living animals could allow to perform more informative preclinical trials with a better evaluation of therapeutic benefit with respect to muscle wasting. We developed a non-invasive procedure based on micro-computed tomography (micro-CT) without contrast agents to monitor hind limb muscle wasting in mouse models of amyotrophic lateral sclerosis (ALS) and cancer cachexia: the transgenic SOD1^G93A^ mouse and the colon adenocarcinoma C26-bearing mouse, respectively. We established the scanning procedure and the parameters to consider in the reconstructed images to calculate the Index of Muscle Mass (IMM). The coefficient of variance for the whole procedure was 2.2%. We performed longitudinally micro-CT scan of hind limbs in SOD1^G93A^ mice at presymptomatic and symptomatic stages of the disease and calculated the IMM. We found that IMM in SOD1^G93A^ mice was lower than age-matched controls even before symptom onset. We also detected a further decrease in IMM as disease progresses, most markedly just before disease onset. We performed the same analyses in the C26-based mouse model losing quickly body and muscle mass because of cancer cachexia. Overall, we found that the reduced muscle content detected by micro-CT mirrored the reduced muscle weight in both disease models. We developed a fast, precise and easy-to-conduct imaging procedure to monitor hind limb muscle mass, useful in therapeutic preclinical trials but also in proof-of-principle studies to identify the onset of muscle wasting. This method could be widely applied to other disease models characterized by muscle wasting, to assist drug development and search for early biomarkers of muscle atrophy. Moreover, reducing the number of mice needed for the experiments and being less distressing are in line with the 3R principle embodied in national and international directives for animal research.

## Introduction

Muscle wasting is a major complication of many chronic diseases, including cancer and neuromuscular disorders, but occurs also, even if more slowly, upon aging (i.e. sarcopenia). It can even lead to death through respiratory failure when involving respiratory muscles, as is the case of amyotrophic lateral sclerosis (ALS) [1]. Thus, preventing it with ad-hoc therapies could result in life extension and amelioration of quality of life in patients afflicted by very diverse diseases, having a great socio-economic impact.

Loss of muscle mass results from imbalanced rates of protein synthesis and degradation. It seems that denervation and/or intrinsic muscle defects (neuromuscular disorders) or persistent systemic inflammation (cancer) causes in skeletal muscles reduced anabolism for less production of growth factors (i.e. IGF-1) and increased catabolism, mainly due to overactivated FOXO3 or STAT3-based pathways [2–4]. Presently, drugs to block this deleterious process are lacking as well as modes to detect it at the earliest, including circulating biomarkers able to predict it in animal models or patients.

ALS primarily affects motor neurons in the motor cortex, brainstem and spinal cord, resulting in progressive muscle atrophy [5]. Cu/Zn superoxide dismutase (SOD1) is the first ALS-causing gene discovered and is associated with 20% of the familial cases and 2–7% of the sporadic cases [6]. Mouse models in which human mutant SOD1 is overexpressed recapitulates several core clinical features of ALS, such as tremors, progressive muscle weakness, locomotor deficits and paralysis, resulting in premature death [7]. These models have been extensively used to dissect pathogenic mechanisms in ALS and to assess efficacy of new therapeutic agents [8,9].

Cancer cachexia is a multi-organ syndrome where the body energy stores are progressively depleted to face the high energy demand of tumor progression. Skeletal muscle is the main tissue to undergo progressive wasting in order to release free amino acids to sustain liver gluconeogenesis and tumor growth [10]. Massive muscle depletion negatively affects the response to therapies, the ability to perform daily activities and precedes death in cancer patients. The most used model to study cancer cachexia is the colon adenocarcinoma C26-bearing mouse because such tumor growth causes fast and progressive body weight loss with signs of muscle wasting, enhanced systemic inflammation (i.e. IL-6) and premature death [11].

Generally, evaluating the degree of muscle atrophy at least in diseased mouse models requires the sacrifice of the animals for muscle dissection and their weighing and/or histological examination. Very often behavioral tests for muscle strength evaluation are used as an outcome measurement in preclinical therapeutic trials. However, the tests normally used are easy to perform serially, but not enough sensitive to detect early muscle changes as disease progresses [12]. Presently, some effort to evaluate non-invasively muscle wasting in living anesthetized animals during the progression of the disease has been done through high-resolution technologies, such as Magnetic Resonance Imaging (MRI) [13–15]. Unfortunately, MRI necessitates of expensive instrumentation, time-consuming image analysis, large dedicated areas in the animal facilities and highly qualified personnel [16]. Low-cost instrumentations, such as dual energy X-ray absorptiometry (DEXA) and Nuclear Magnetic Resonance Relaxometry (EchoMRI), may estimate whole body composition [17]. However, it remains to be determined whether they are enough sensitive to monitor *in vivo* the progression of diseases affecting specific muscles, such as neuromuscular diseases.

Micro-computed tomography (micro-CT) is an accurate X-ray based imaging technology for small animal models. The maintenance costs of micro-CT are significantly lower than MRI and the former does not require highly trained personnel [16]. It is commonly used for skeletal imaging because bones are densely mineralized tissues with excellent X-ray attenuation properties [18]. Nonetheless, micro-CT is also used for imaging of soft-tissues, blood vessels, and visceral organs in association with X-ray opaque contrast agents. However, the contrast agents currently available for small laboratory animals have poor contrasting properties or are toxic [19]. Herein, we propose a novel micro-CT-based method to image mouse hind limb muscles without contrast agents through a fast, precise and easy-to-conduct procedure. We measured longitudinally the hind limb muscle mass of mice afflicted by diseases resulting in muscle wasting in a short- (cancer cachexia) and long-term (ALS) experimental setting. In both disease models, we were able to appreciate a reduced muscle content by micro-CT that mirrored the reduced muscle weight of euthanized mice.

## Materials and methods

### Ethics statement

Procedures involving animals and their care were conducted in conformity with the following laws, regulations, and policies governing the care and use of laboratory animals: Italian Governing Law (D.lgs 26/2014; Authorisation n.19/2008-A issued March 6, 2008 by Ministry of Health); Mario Negri Institutional Regulations and Policies providing internal authorization for persons conducting animal experiments (Quality Management System Certificate–UNI EN ISO 9001:2008 –Reg. No. 6121); the NIH Guide for the Care and Use of Laboratory Animals (2011 edition) and EU directives and guidelines (EEC Council Directive 2010/63/UE). The Statement of Compliance (Assurance) with the Public Health Service (PHS) Policy on Human Care and Use of Laboratory Animals has been recently reviewed (9/9/2014) and will expire on September 30, 2019 (Animal Welfare Assurance #A5023-01). The Mario Negri Institutional Animal Care and Use Committee and the Italian Ministry of Health (Direzione Generale della Sanità Animale e dei Farmaci Veterinari, Ufficio 6) prospectively reviewed and approved the animal research protocols of this study (prot. n. 9F5F5.41.EXT.9 and 9F5F5.59) and ensured compliance with international and local animal welfare standards. Animals were bred and maintained at the IRCCS-Istituto di Ricerche Farmacologiche "Mario Negri", Milano, under standard conditions, temperature 21±1°C, relative humidity 55±10%, 12 hour-light schedule, food and water *ad libitum*. Euthanasia was performed by exposure to CO_2_ and then by cervical dislocation in home cages. No animals died without euthanasia due to the experimental procedures. All research staff involved in the experiments were authorized to handle animals after a special Institutional course in animal care and use.

### Animal models

The ALS mouse model, B6SJL-Tg-SOD1-G93A-1Gur, expressing ~ 20 copies of mutant human SOD1 with a Gly93Ala substitution (SOD1^G93A^), was originally obtained from Jackson Laboratories and maintained on a C57BL/6JOlaHsd (Harlan Inc., Indianapolis, IN, USA) background at the IRCCS-Istituto di Ricerche Farmacologiche "Mario Negri". This transgenic SOD1^G93A^ mouse strain starts to show muscle strength and motor function impairment at 125 ± 6 (mean value ± SD) days of age (18 weeks) and survives up to 180 ± 11 days of age (26 weeks), as previously described [20,21]. SOD1G93A transgenic mice were identified by PCR on DNA from tail biopsies, as previously reported [22]. Mice were monitored twice a week up to 22 weeks of age and then every day. Euthanasia was performed soon after mice failed to right themselves within 30 s of being placed on their side.

Murine colon adenocarcinoma C26 cells [11] were kindly donated by Prof. Mario Paolo Colombo (IRCCS-Istituto Nazionale dei Tumori, Milan) and cultured in DMEM high-glucose (Gibco) with 10% FBS and 1mM L-glutamine (Gibco) as in [23]. 1*10^6^ cells in 200 ul of PBS were subcutaneously (s.c.) inoculated on the right flanck of 9 week-old BALB/c male mice Harlan Laboratories, while control group were injected with equal amount of PBS. Tumor growth was measured manually twice a week with Vernier Caliper, while cachexia was followed by recording body weight. Mice were monitored twice a day and were usually sacrificed soon after body weight loss reached > 20% consecutively for 72 hours and/or the animals showed at least four out of five clear signs of distress (ruffled fur, tremor, loss of mobility, kyphosis, anorexia), however with a tumor not exceeding 10% of body weight.

### Micro-CT

**Fig 1:**
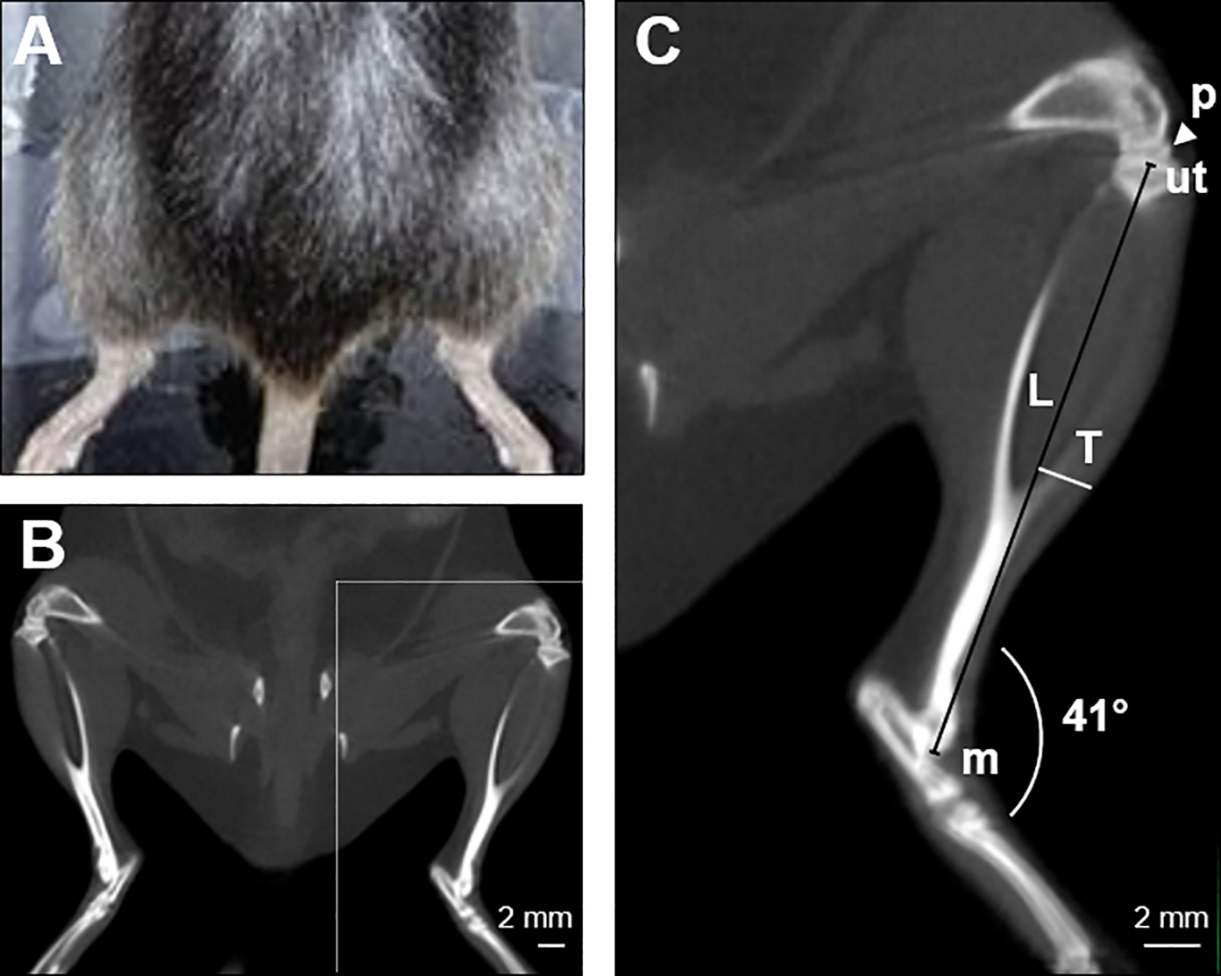
Micro-CT evaluation of the hind limb muscle mass and definition of the index of muscle mass (IMM). (A) Mice were placed prone on the micro-CT bed leaving the hind limbs in a natural position (foot and tibia angle mean ± SD: 41° ± 6, n = 30). (B) Scanned raw images were reconstructed in 3D and analyzed using Micro View analysis software. Scale bar, 2 mm. (C) For the evaluation of the muscle mass, the following parameters from 3D reconstructed images were measured: the distance from the upper extremity of the tibia (ut) to the medial malleolus (m), defined as the length of the tibia (L), and the perpendicular distance from the half-length of the tibia to the external margin of the hind limb muscle, referred to as the thickness of the muscle (T). These measurements were assessed on the plane in which the patella (p) and the upper extremity of tibia (ut) were clearly visualized. Scale bar, 2 mm.

Micro-CT was performed with an Explore Locus micro-CT scanner (GE Healthcare) without contrast agents. Before the analysis, mice were anesthetized with a continuous flow of 3% isofluorane/oxygen mixture and placed prone, leaving the hind limbs on a natural position (foot and tibia angle mean ± SD: 41° ± 6, n = 30), on the micro-CT bed (Fig 1A). A micro-CT lower resolution (Bin-4) protocol was performed using 80 kV, 450 μA with 100 msec per projection and 400 projections over 360° for a total scan time of 10 minutes, as previously described [18]. The isotropic resolution of this protocol is 93 μm. The scanned images were reconstructed in 3D and analyzed using Micro View analysis software (version 2.1.1; GE Healthcare). The following parameters were measured (Fig 1B and 1C): the distance from the upper extremity of the tibia to the medial malleolus (length of the tibia, L); the perpendicular distance from the half-length of the tibia to the external margin of the hind limb muscle (thickness of the muscle, T). These measurements were acquired by keeping the z axis fixed on the plane where the patella and the upper extremity of tibia were clearly identified. The Index of Muscle Mass (IMM) was defined as the ratio between T and L. Cross-sectional area of the muscle group at half length of the tibia was also measured using advanced ROI contour tool in Micro View analysis software. For each mouse, the left and right leg were analyzed and averaged.

### Muscle and body weight

Muscle weight: Gastrocnemius (GC) and Tibialis Anterior (TA) were freshly dissected, as previously described [24,25], and immediately weighed on an analytical balance with a readability of 0.1 mg and a linearity of ± 0.2 mg (Crystal series, Gibertini). Body weight: mice were weighed twice a week on a balance with a readability of 0.1 g and a linearity ± 0.3 g (EU-C 7500PT, Gibertini).

### Grip strength test

Grip strength in the SOD1^G93A^ mice and controls was evaluated essentially as previously described [22]. Briefly, animals were placed on a horizontal grid and the tail was gently pulled until they grabbed the grid with their fore and hind paws. The grid was then gently turned upside down and the latency time of the mouse to fall was recorded for a maximum of 90 sec. The test was repeated three times and the maximal time of latency was plotted. The eventual fall occurs on a surface of polystyrene covered with sawdust from a distance of 20 cm.

Grip strength in C26-bearing mice and controls was evaluated by a dynamometer (Basile), as previously described [26], with some modifications. Briefly, the mouse was lifted by the tail and allowed to grasp with its fore paws a horizontal bar, which is connected to a mechano-electric transducer. After a four-day test training, mice were injected subcutaneously with the tumor (or PBS) and grip strength was measured almost daily after tumor injection. The grip strength was measured at times when the mouse released the horizontal bar as a result of a gentle traction applied by the operator. The test was repeated three times per mouse and the best trial recorded (equivalent to the maximal force) was used for data analyses.

### Statistical analysis

Experiments with the ALS mouse model: for the grip strength and body weight analysis, two-way ANOVA for repeated measures followed by Bonferroni’s *post-hoc* test was applied for the comparison between SOD1^G93A^ mice and controls. For muscle weight and IMM analysis at different stages of the disease, one-way ANOVA followed by Newman-Keuls multiple comparison test was applied for the comparison between the different groups. Experiments with the cachexia mouse model: for grip strength, body weight and IMM analysis, two-way ANOVA for repeated measures followed by Bonferroni’s *post-hoc* test was applied for the comparison between C26-bearing mice and controls. For muscle weight and IMM analysis at 12 days after tumor inoculation, Student’s t-test was applied for the comparison between C26-bearing mice and controls. Unpaired, two-tailed Student’s t test was used to compare the performance of two operators in IMM measurements. Pearson’s correlation coefficient was calculated to measure the strength of the association between the mean values of the muscle weight and the IMM. Analyses were performed using GraphPad Prism software (GraphPad Software, San Diego, CA, USA), version 7.02 and Statview Software (StatView for WindowsSAS Institute Inc., Version 5.0.1, Cary, NC, USA).

## Results

### Longitudinal evaluation of mouse hind limb muscle mass by non-invasive micro-CT analysis

We explored the possibility to evaluate longitudinally the hind limb muscle mass through non-invasive micro-CT technology. To develop a method that could be at once safe and accurate for living animals, a compromise between the X-ray exposure and spatial resolution was reached. In mice the median lethal dose is reported to range between 5–8 Gy depending on age, sex and strain [27–29]. In animals undergoing multiple sequential scans, exposures must be kept low, since the effects of sublethal radiation doses can be added together [30]. Using our micro-CT protocol, the image acquisition time was 10 min and the radiation dose was 0.11 Gy [18]. We therefore considered measures of easily recognized structures, that need low X-ray exposure, such as bones and the hind limb margin at the interface with air, to derive an index that reflects hind limb muscle mass (IMM). To test the consistency of these measurements, we calculated the coefficient of variance (CV) of repeated measurements of IMM in the same mouse at three different times (T1, T2, T3). We used 6 nontransgenic mice (#1–3 C57BL/6JOlaHsd and #4–6 BALB/c) that were analyzed by two different operators, A and B (Table 1). We had in all cases a CV <5%, a 2.2% mean CV and no significant difference between the two operators (mean CV for operator A = 2.9% and for operator B = 2.3%) by Student’s t test, indicating that the procedure is highly reproducible and repeatable. We also tested the cross-sectional muscle area at half length of the tibia (as typically done in humans), but such parameter had a higher CV (9%). This was probably due to the limited spatial resolution obtained with our micro-CT scanning protocol. Indeed, measurements of cross-sectional area of muscle groups by micro-CT could be a valid option using higher exposures [31]. However, using the lowest possible exposure avoids any potential detrimental effects of radiation [32], and this aspect is important to consider especially for mouse models with already severe symptoms subjected to multiple scanning procedures. We next measured IMM in mice suffering from ALS or cancer cachexia during disease progression to establish possible IMM correlation with hind limb muscle weight.

**Table 1:** CV of repeated measurements of IMM in the same mouse at three different times.

Mouse^a^	Operator	T1^b^	T2^b^	T3^b^	CV^c^
1	A	0,159	0,162	0,147	4,9
2	A	0,166	0,159	0,164	2,3
3	A	0,164	0,164	0,168	1,5
4	B	0,145	0,156	0,156	4,2
5	B	0,158	0,157	0,157	0,5
6	B	0,146	0,152	0,147	2,1

^a^Mouse strains were: C57BL/6JOlaHsd (1–3), BALB/c (4–6).

^b^T1-T3: for mice 1–3 T1 = baseline, T2 = 21 days, T3 = 42 days; for mice 4–6 T1 = baseline, T2 = 3 days, T3 = 6 days.

^c^CV is expressed as %.

### Non-invasive micro-CT analysis of hind limb in SOD1^G93A^ mice during disease progression

**Fig 2:**
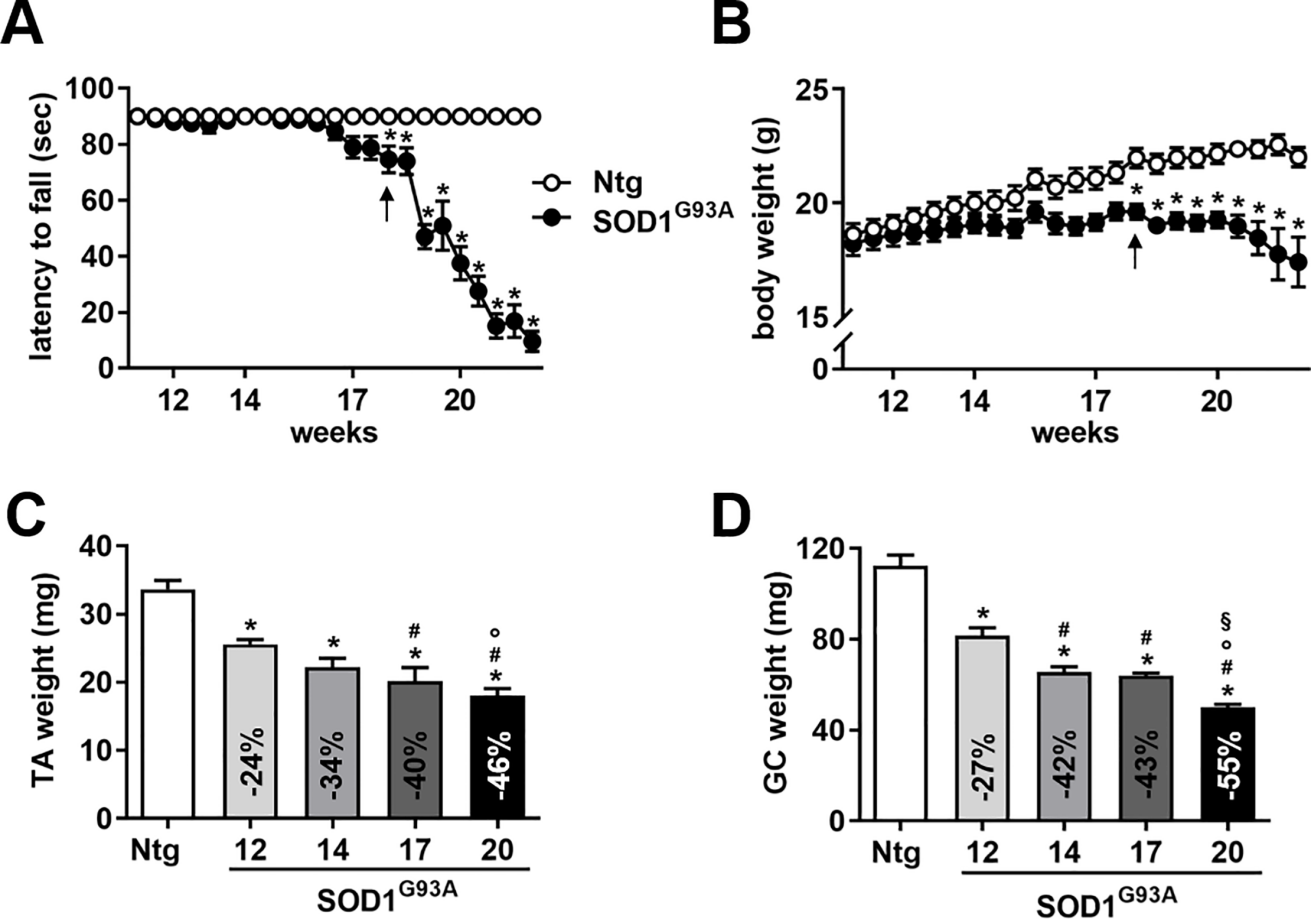
SOD1^G93A^ mice have a lower hind limb muscle mass than Ntg controls already at a presymptomatic stage of the disease. (A-B) Grip strength as latency to fall (sec) and body weights (g) in Ntg and SOD1^G93A^ mice are reported from 11 to 22 weeks of age (n = 10 per group). Data are expressed as mean ± SEM. *, p < 0.05 by two-way ANOVA for repeated measures (the interaction between genotype and time was significant in A and B with p < 0.0001), Bonferroni’s post-hoc test. (C-D) The weight of TA (C) and Gastrocnemius (GC) (D) was measured in Ntg and SOD1^G93A^ mice at 12, 14, 17 and 20 weeks of age (n = 5 for each group). The weight of TA and GC was lower in SOD1^G93A^ mice than age-matched Ntg controls already at a presymptomatic stage of the disease (12 weeks of age). Muscle weight was expressed in mg. Decrease in % was reported with respect to Ntg mice at 12 weeks of age. Data (mean ± SEM) were significantly different (p < 0.05), by one-way ANOVA, Newman-Keuls *post-hoc* test: *, *versus* Ntg; #, *versus* 12-week old SOD1^G93A^; °, *versus* 14-week old SOD1^G93A^;§, *versus* 17-week old SOD1^G93A^ mice.

Disease progression in SOD1^G93A^ mice is usually monitored by behavioral tests, such as grip strength, and body weight assessment. Fig 2A and 2B reports grip strength and body weights of SOD1^G93A^ mice and nontransgenic (Ntg) littermates from 11 to 22 weeks of age, respectively. The first clinical signs of motor dysfunction, as latency to fall from a grid turned upside down, were observed from 18 weeks of age (mean ± SEM: 75 ± 7 in SOD1^G93A^ mice *versus* 90 ± 0 in control mice, p<0.05) (Fig 2A). The difference in mean body weight (g) between SOD1^G93A^ mice and control mice was also significant from 18 weeks of age (mean ± SEM: 20 ± 0.4 in SOD1^G93A^ mice *versus* 22 ± 0.4 in control mice, p<0.05). Interestingly, when we measured the weights of the biggest hind limb muscles, TA and GC, we could detect significant muscle wasting in SOD1^G93A^animals with respect to age-matched controls already at 12 weeks of age (Fig 2C and 2D), at a presymptomatic stage of the disease. More in detail, we found that muscle weights of TA and GC of SOD1^G93A^ mice were reduced by 24% and 27%, respectively. At 20 weeks of age, at an advanced stage of the disease, the muscle content of SOD1^G93A^ mice was further decreased, down to 46% and 55% for TA and GC, respectively (Fig 2C and 2D).

**Fig 3:**
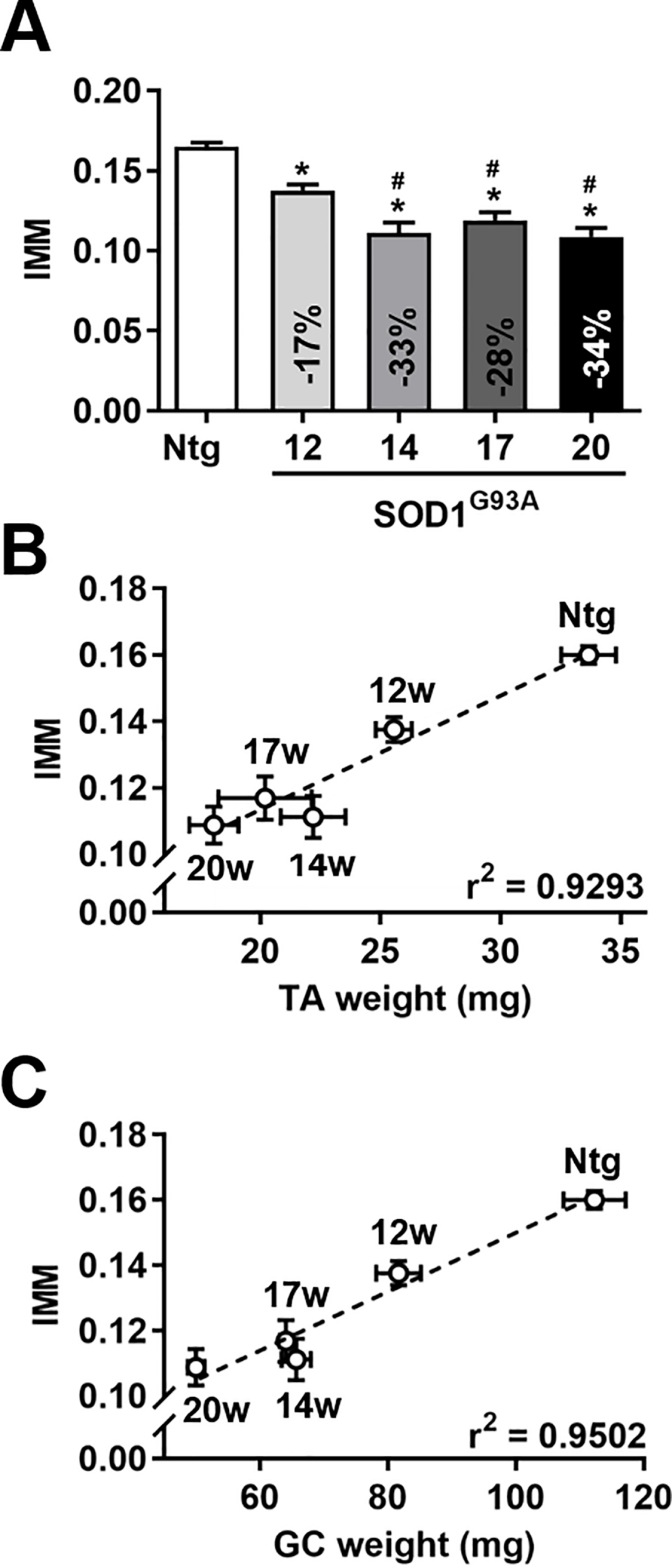
IMM correlates with hind limb muscle weight in SOD1^G93A^ mice as disease progresses. (A) IMM was evaluated in Ntg and longitudinally in SOD1^G93A^ mice at 12, 14, 17 and 20 weeks of age (n = 5 for each group). Decrease in % was reported respect to Ntg mice at 12 weeks of age. Data, expressed as mean ± SEM, were statistically significant different (p < 0.05), by one-way ANOVA, Newman-Keuls *post-hoc* test: *, *versus* Ntg; #, *versus* 12-week old SOD1^G93A^ mice. (B-C) A correlation analysis was done with the mean values of the IMM and of the muscle weight of TA (B) and Gastrocnemius (GC) (C) for the different experimental groups. The analysis confirmed a significant (p < 0.008 for TA, p < 0.005 for GC) direct correlation between the two type of measurements with r^2^ = 0.9293 and r^2^ = 0.9502 for TA and GC respectively. Data are expressed as mean ± SEM.

We performed longitudinally micro-CT scans of hind limb muscles in SOD1^G93A^ mice at 12, 14, 17 and 20 weeks of age and calculated the IMM, as previously described. Consistently, we found that IMM in SOD1^G93A^ mice was lower (-17%) than age-matched controls already at 12 weeks of age (Fig 3A). We also detected a further decrease in IMM as disease advances, most markedly between week 12 and 14, but not between week 14 and 17, as occurs for both muscle weights (Fig 2C and 2D). At 20 weeks of age, the IMM of SOD1^G93A^ mice decreased by 34% compared to controls, a less prominent reduction than that detected by muscle weight. Of note, the muscle content of nontrangenic mice did not change between 12–20 weeks of age, either if we consider the muscle weight or IMM (data not shown). We next examined whether there was a correlation between IMM and TA and GC muscle weights as disease progresses (Fig 3B and 3C). Importantly, we found that IMM significantly correlated with both muscle weights, with a Pearson's correlation coefficient > 0.9 and a slightly higher value for GC. We finally analyzed correlation between IMM and muscle weights within the same cohort of animals (n = 10, 5 nontransgenic mice and 5 SOD1^G93A^ mice at 12 weeks of age). Correlation was significant (p = 0.003) only for GC with a Pearson's correlation coefficient = 0.7. Instead, there was no correlation between the cross-sectional muscle area at half length of the tibia and muscle weight (data not shown). Overall, we believe that this method could be valuable to detect muscle atrophy in a mouse model of ALS during the progression of the disease, especially in the early stages of the disease.

### Longitudinal non-invasive micro-CT analysis of hind limb in mice undergoing C26-induced cachexia

**Fig 4:**
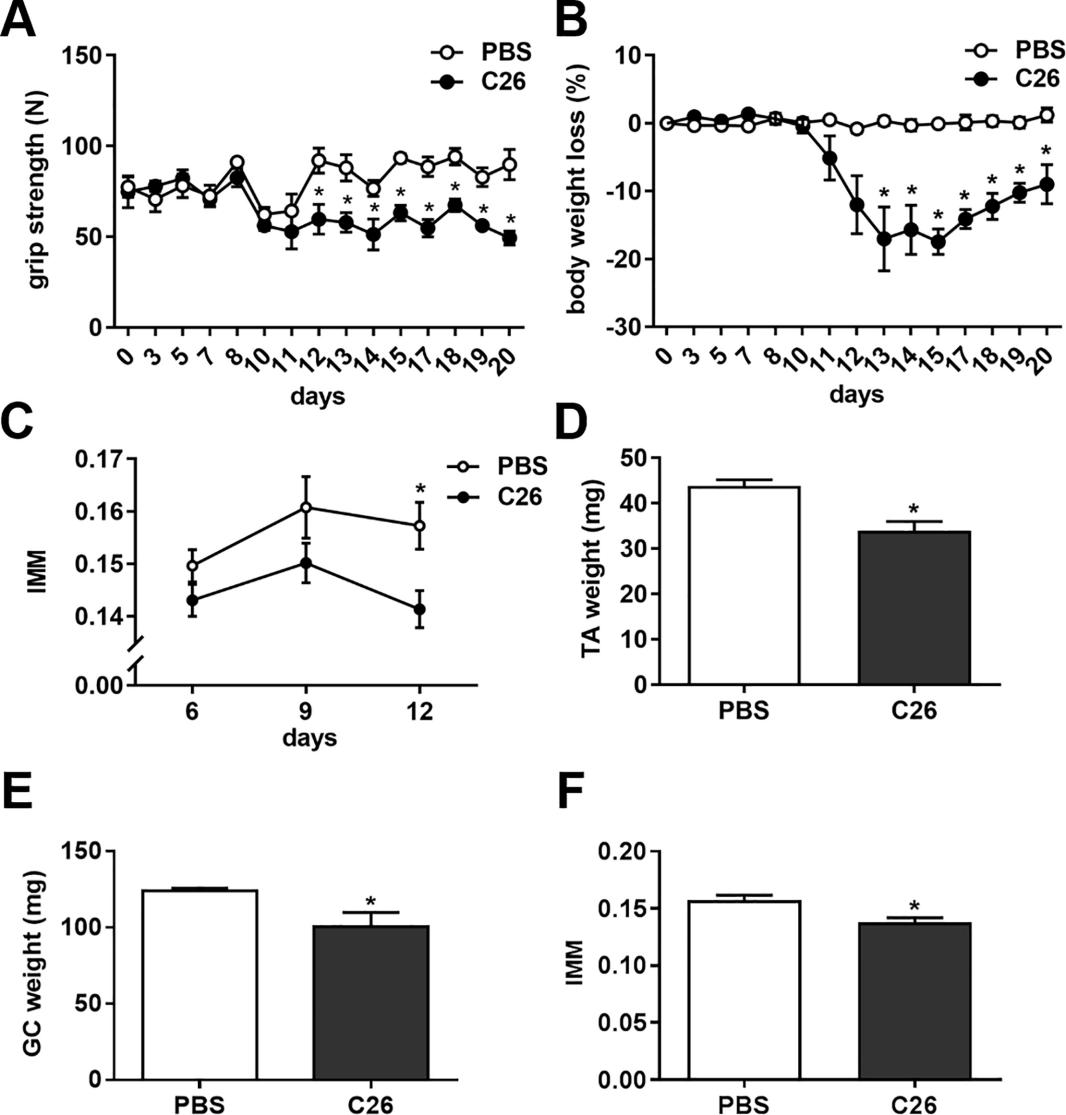
C26-bearing mice display hind limb muscle mass loss at 12 days from tumor injection. (A-B) Grip strength (N) and body weight loss (%) are shown for C26-bearing mice and PBS-injected ones (n = 3 and n = 4, respectively). Data are expressed as mean ± SEM. *, p < 0.05 by two-way ANOVA for repeated measures (the interaction between treatment and time was significant in A with p = 0.0002 and in B with p < 0.0001), Bonferroni’s *post-hoc* test. (C) Muscle mass loss evaluated by micro-CT according to IMM definition in C26-bearing mice and PBS-injected ones (n = 11 and n = 4, respectively). Data are expressed as mean ± SEM. *, p < 0.05 by two-way ANOVA for repeated measures, Bonferroni’s *post-hoc* test. (D-E) Muscle weights of TA (D) and GC (E) of C26-bearing mice and PBS-injected ones are reported 12 days after tumor inoculation (n = 4). Data are expressed as mean ± SEM. * p < 0.05 by Student’s t-test, *versus* PBS. Both muscle weights are expressed in mg. (F) A similar result was obtained by evaluating the IMM as an indicator of muscle mass in the same set of mice (n = 4). Data are expressed as mean ± SEM, and were statistically significant different (p < 0.05) by the Student’s t-test.

Cachexia progression in C26-carrying mice was monitored by dynamometer grip strength test and body weight assessment, as usually done [33]. Fig 4A and 4B reports grip strength and body weight loss of C26-bearing mice and PBS-injected ones as control, until 15 days from tumor injection, when two of them had to be euthanized because of clear signs of distress [23]. Twelve days after tumor injection, C26-bearing mice already showed a drastic decrease in forelimb strength (N) (mean ± SEM: 60 ± 7 in C26-bearing mice *versus* 92 ± 7 in PBS-injected mice, p<0.05) almost in concomitance with a dramatic body weight loss starting 13 days from tumor injection (mean ± SEM: -17% ± 3.6 in C26-bearing mice *versus* -0.3% ± 0.6 in PBS-inoculated mice, p<0.0001). Interestingly, when we measured longitudinally the hind limb muscle mass of C26-carrying mice with the above-described micro-CT method (Fig 4C), we found a tendency to lose muscle mass already 9 days from tumor injection, at times when the fore limb grip strength and body weight were unaffected. Loss of muscle mass measured as IMM of C26-bearing mice increased and became different from PBS-injected ones at 12 days from tumor injection (Fig 4C). On day 12, we compared the weights of TA (Fig 4D) and GC (Fig 4E) of four randomly-chosen C26-bearing mice and PBS-injected ones. The weights of both muscles were decreased by 23% in TA and by 20% in GC of C26-bearing mice (p≤0.05). Intriguingly, the decrease in muscle mass of C26-bearing mice (Fig 4D and 4E) was similar to the one obtained through the non-invasive micro-CT method described (14%, p<0.05) in the same animals (Fig 4F). So, we confirmed this method as useful to detect muscle atrophy in another unrelated mouse model, displaying muscle wasting upon cancer growth.

## Discussion

We here report a new method to monitor hind limb muscle mass in living mouse models of ALS and cancer cachexia that display muscle wasting during disease progression. It employs a micro-CT-based instrumentation, anesthetized animals, no contrast agents, short scan time, and fast data analysis. Micro-CT is an imaging technology that allows to acquire detailed anatomical structures with excellent spatial resolution in small rodents. However, the lack of structural contrast of soft tissues, in absence of specific agents, has mainly restricted its application to the bone. In fact, CT contrast agents currently used for humans are not applicable to rodents because not compatible with micro-CT timing of even the best preclinical imagers, for their rapid renal clearance [19]. Importantly, we show that the IMM parameter we derived from hind limb micro-CT images, acquired without contrast agents and mostly relying on skeletal imaging, strongly correlates with muscle weights of either TA or GC obtained from euthanized mice. Our protocol, employing low X-ray exposures, is safe and especially recommended for multiple and frequent imaging of mouse models with severe diseases. Moreover, such method may reduce the number of mice needed for the experiments (Reduction) and is less distressing for animals than behavioral tests (Refinement), in agreement with the 3R principle embodied in national and international directives for animal research. Notably, CT is already recommended as one of the “gold standard” imaging techniques to evaluate age-related modifications of skeletal muscle in clinical trials [34]. Monitoring muscle loss in living animals by micro-CT could allow to perform more informative preclinical trials with a precise evaluation of therapeutic benefit with respect to muscle wasting.

In the last years, several evidence indicate that the pathological process in ALS is extending beyond motor neurons. Other cell types, including muscle fibers, and factors, also outside the central nervous system, are probably contributing to the pathology [24,35]. Studies in the mutant SOD1 mice have shown that neuromuscular junction destruction followed by severe axonal degeneration precedes motor neuron cell body loss and is one of the first pathological events [36,37]. Proteomic, transcriptomic and energy metabolism alterations in hind limb muscles have been detected well before the onset of clinical symptoms [38–40]. Muscle-restricted mutant SOD1 expression causes muscle damage with an inflammatory response in the spinal cord and later on also motor neuron loss [41,42]. On the other hand, neuroprotective therapies that could rescue motor neuron from apoptosis had limited effect on muscle denervation and survival [43,44]. It is therefore important to consider the specific action on muscles of potential new drugs when developing a therapeutic approach for ALS. Using such micro-CT method, we found that reduced muscle content in a SOD1^G93A^ mouse model of ALS anticipated by at least 6 weeks the clinical onset measured by body weight loss and functional tests, in agreement with a previous longitudinal study using MRI [14]. We also observed that after an initial substantial loss of muscle mass, there was an apparent arrest in the process of muscle wasting, before the clinical onset, that was also confirmed by muscle weight measurements. This probably reflects a phase of compensatory re-innervation that was already reported in the mutant SOD1 mouse at that stage of the disease [36,45]. At the latest time point analyzed, which corresponds to an advanced stage of the disease, IMM seems to be less sensitive than muscle weight to unravel atrophy.

Overall, these observations indicate that micro-CT scan of hind limb is sensitive enough to monitor the course of the disease, especially in the early stages. Moreover, it underlines the limitations of providing a treatment when the pathological process has already started. On the other hand, several clinical trials failed after apparent promising results in the mouse model treated before the onset of clinical symptoms. Unfortunately, presymptomatic treatments could be applicable only to individuals at-risk, carriers of ALS-associated gene mutations, and not to the majority of ALS patients with unknown etiology. In line with this, it would be interesting to look more in detail at the prodromal phase in the mouse model to identify early biomarkers of muscle wasting that can be then validated in individuals at risk of developing ALS.

The most recent definition of cancer cachexia refers to it as a "multifactorial syndrome characterized by an ongoing loss of skeletal muscle mass (with or without loss of fat mass) that cannot be fully reversed by conventional nutritional support and leads to progressive functional impairment" [46]. Assessment of lean body mass through CT scan is already applied in cancer patients to identify muscle wasting and it is much more sensitive than changes of body mass index or waistline [47,48], which cannot detect muscle atrophy in obese or overweight cancer patients [49] as well as sarcopenia in fat and old individuals. Despite the fact that cancer often causes wasting of multiple organs (muscles, fat, heart and bones), only depletion of skeletal muscle tissues mostly predicts bad prognosis in patients [50,51]. As such, following muscle wasting with *in vivo* imaging-technology provides a unique opportunity to understand when and how this condition becomes life-threatening [52]. Similarly, the C26 based-model undergoes cachexia with wasting of multiple organs (muscles, fat, heart and bones) [53,54], but only recently some of them have been successfully characterized by us and others also through *in vivo* imaging technology. The diaphragm muscle has been imaged and found reduced in size and function [55] as well as the heart through echocardiography [56] and the bone and fat through dual energy X-ray absorptiometry or micro-CT [57,58]. However, none of these studies performed a longitudinal monitoring of the analyzed organ by *in vivo* imaging technology, but only at one predefined timepoint, being even less informative than the weighed dissected tissue. It is time to apply a combination of these techniques in a longitudinal setting to understand which is the first organ to display morphological or functional aberrations in response to cancer growth, clarifying the chronological order of the pathological events and eventually suggesting possible cause-and-effect relationships. Although not as clearly as in the ALS mouse model, because of the very rapid progression of C26-related cachexia, we did see a tendency in IMM dropping already 9 days after tumor injection, anticipating reduced body weight loss and grip strength. Noteworthy, in such an experimental design, where three micro-CT scans per mouse per week were performed, we observed no signs of distress in mice.

In conclusion, we developed an imaging procedure that could be implemented in therapeutic preclinical trials to monitor hind limb muscle mass but also in proof-of-principle studies to identify the onset of muscle wasting. Knowing exactly the timing of muscle loss will allow to plan more accurately the schedule for drug administration and blood withdrawal from mice for the search of early biomarkers of muscle atrophy during various diseases. We believe this procedure could be widely applied to other disease models involving muscle wasting, including aging animals, as well as to other species, as rats, to assist drug development and search for early biomarkers of muscle atrophy.
